# Advanced glycation end-products stimulate basic fibroblast growth factor expression in cultured Müller cells

**DOI:** 10.3892/mmr.2012.1152

**Published:** 2012-10-24

**Authors:** JING AI, YAO LIU, JUN-HUI SUN

**Affiliations:** 1 Department of Ophthalmology, Second Affiliated Hospital (Binjiang Branch), School of Medicine, Zhejiang University, Hangzhou, Zhejiang 310009; 2 Department of Ophthalmology, Zhongda Hospital, Southeast University, Nanjing, Jiangsu 210009; 3 Key Laboratory of Combined Multi-organ Transplantation, Ministry of Public Health, First Affiliated Hospital, School of Medicine, Zhejiang University, Hangzhou, Zhejiang 310003, P.R. China

**Keywords:** diabetic retinopathy, advanced glycation end-products, basic fibroblast growth factor, Müller cells

## Abstract

Accumulating evidence points to a causal role for advanced glycation end-products (AGEs) in the development of diabetic vascular complications, including diabetic retinopathy (DR). To assess the reciprocal correlation between AGEs and basic fibroblast growth factor (bFGF), the effects of AGEs on the production of bFGF by Müller cells were investigated. Müller cells were cultured from adult rabbit retinas. The AGEs were prepared with highly glycated bovine serum albumin (BSA) and the control non-glycated BSA (BSA control) was incubated under the same conditions without glucose. Cultured Müller cells were exposed to AGEs or BSA control (volume percentages were 4, 8, 16, 32 and 64%) for a time course of 1, 3, 6 and 9 days in their desired medium. The expression of bFGF in Müller cells was evaluated by immunocytochemistry. Quantification was performed by densitometry using computerized image analysis with dedicated software. AGEs in a volume percentage of 16 and 32% on day 1 and in a volume percentage of 16, 32 and 64% on days 3, 6 and 9 increased the bFGF expression in Müller cells (P<0.05). Additionally, AGEs upregulated bFGF expression in Müller cells in a time-dependent manner. In conclusion, the treatment of Müller cells with AGEs resulted in a dose- and time-dependent elevation of bFGF in the culture medium. The results from this study suggest that the increased formation of AGEs in the vitreous may be involved in the development of DR by inducing the production of bFGF by retinal Müller cells.

## Introduction

Diabetic retinopathy (DR) still remains the leading cause of blindness worldwide. Consequently, there is a need for further investigation of the pathogenesis of DR to develop better more efficient therapeutic techniques. A considerable amount of evidence supports a causal role for advanced glycation end-products (AGEs) in the development of diabetic complications. AGEs have diverse biological properties, which include protein-linking, cellular activation, growth promotion and induction of vascular dysfunction (1,2). They represent an integrated measure of glucose exposure over time (3,4), increase in diabetic retinas and correlate with the onset and severity of retinopathy (5).

Müller cells, the predominant glial cells of the retina, express a diversity of ion channels and are responsive to numerous growth factors, cytokines and neurotransmitters. Molecules in the microenvironment regulate Müller cell function, structure, location, number and intercellular interactions. Müller cells themselves are sources for molecules that regulate these glia (6). It has been confirmed that these factors and cytokines are known to be involved in the pathogenesis of DR. The molecules include basic fibroblast growth factor (bFGF) (7), vascular endothelial growth factor (VEGF) (8,9), insulin-like growth factor (10), transforming growth factor-β (11), hepatocyte growth factor (HGF) (12) and pigment epithelium-derived factor (PEDF) (13). The angiogenic cytokines contribute to the regulation of endothelial cell proliferation, migration, extracellular proteolysis by matrix metalloproteinases (MMPs) and vessel wall remodeling (14).

VEGF and bFGF (15) are two of the most important pro-angiogenic cytokines and VEGF may be associated with the breakdown of this blood-retinal barrier. In contrast to VEGF, bFGF has broader biological functions in the development of intraocular neovascularization in DR (16,17). Although VEGF expression is induced by AGEs (11,18), it remains unclear whether AGEs initiate the production of bFGF by Müller cells during the early stages of DR.

In the current study, we performed specific experiments to examine the correlation between AGEs and the expression of bFGF in cultured Müller cells *in vitro* in order to further explore the mechanisms behind DR.

## Materials and methods

### Cell cultures

The Müller cell culture was prepared as described previously (19,20). Briefly, in phosphate-buffered saline (PBS) with 100 U/ml penicillin, 100 mg/ml streptomycin solution, the eye of an adult New Zealand white rabbit weighing 2.0–2.5 kg was cut 2 mm away from the limbus circumferentially and then the anterior segment and vitreous were removed and discarded. The retina was carefully detached and the avascular non-medullated fraction was removed in order to prevent contamination by astrocytes and oligodendrocytes. The residual retina was cut into 0.5×0.5-mm pieces under a biomicroscope and the fragments were centrifuged at 150 × g for 5 min. The tissue pieces were then gently dissociated by pipetting up and down and were planted in a 25-cm^3^ culture flask using Dulbecco’s minimum essential medium (DMEM) (Gibco-BRL, Grand Island, NY, USA) supplemented with 20% fetal bovine serum (FBS; SAFC, St. Louis, MO, USA), 100 U/ml penicillin, 100 mg/ml streptomycin and cultured at 37°C in a humidified incubator saturated with 5% CO_2_ and 95% air. Almpost all the explants adhere to the surface of the flask within 7 days. Previous immunocytochemical and electron-microscopic studies had confirmed the high purity of Müller cell cultures (19). After reaching 80–100% confluence, the cells were trypsinized and subcultured onto glass coverslips at an approximate density of 1×10^5^/cm^2^ cells. Passage 1 was used for all experiments.

### Preparation of AGEs

Bovine serum albumin (BSA) (fraction V, Sigma Chemical Co., St. Louis, MO, USA) was glycated by incubation with 0.5 M glucose at 37°C for 6–12 weeks under anaerobic and sterile conditions, as described previously (21). Control non-glycated BSA (BSA control) was incubated under the same conditions without glucose. At the end of the incubation period, samples were extensively dialyzed against PBS to remove unbound sugars. Dialyzed glycated protein was characterized based on fluorescence at 446 nm upon excitation at 360 nm using a fluorescence spectrometer (model LS-3B, Perkin-Elmer Corp., Norwalk, CT, USA). Endotoxin content in each sample was determined by the Limulus amebocyte lysate assay (E-Toxate, Sigma Chemical Co.) and was consistently found to be below detectable levels (<0.2 ng/ml).

### Immunohistochemistry analysis of bFGF secretion

Müller cells were separately exposed to AGEs (volume percentage was 0, 4, 8, 16, 32 and 64%) in DMEM supplemented with 10% FBS. The BSA control was used under similar conditions as the control. Samples were incubated at 37°C in a humidified incubator saturated with 5% CO_2_ and 95% air for 1, 3, 6 and 9 days. Conditioned medium without AGEs or BSA control was used as the blank control.

Unless otherwise stated all washes were for 3×5 min in PBS at pH 7.4 and were performed at room temperature while incubations were at 37°C. Sections were brought to room temperature on days 1, 3, 6 and 9 separately, washed, then fixed by immersion in acetone (4°C) (Wuhan Boster Biological Technology, Ltd., Wuhan, China) for 10 min. The slides were air-dried. Endogenous peroxidase activity in the biopsy specimen cryostat sections was blocked with 3% H_2_O_2_ for 30 min and then the slides were washed and placed in 5% BSA confining liquid for 20 min. The specimens were incubated at 4°C overnight with a rabbit anti-rabbit polyclonal antibody against bFGF (Wuhan Boster Biological Technology, Ltd.) at a dilution of 1:200. The negative controls were exposed to the secondary antibody only and processed as described above. The sections were washed and then incubated with biotinylated goat anti-rabbit secondary antibody (SABC-POD kit, Wuhan Boster Biological Technology, Ltd.) for 20 min. Subsequently, a tertiary layer of streptavidin peroxidase was applied according to the manufacturer’s instructions (SABC-POD kit, Wuhan Boster Biological Technology, Ltd.). Antigen-antibody complexes were detected by incubation with diaminobenzidine (DAB) (Wuhan Boster Biological Technology, Ltd.) at room temperature for 10 to 30 min. Then slides were lightly counterstained with Mayer’s hematoxylin (Wuhan Boster Biological Technology Ltd.) for 30 sec. Positive cells were brown-stained, and non-brown-stained cells were considered negative. Finally, the sections were washed, dehydrated, embedded in paraffin and photographed.

### Quantitative immunohistochemical analysis

For immunocytochemical analysis, sections were coded and counted in a blind fashion by using a light microscope (Axioscop, Zeiss, Jena, Germany). A total of 6 visual fields from randomly selected areas in the sample coverslips were examined. Scopes were chosen as the percentage of angiogenic factor-positive cells that colocalized with bFGF. Immunohistochemistry staining gray scale was analyzed by Image-Pro Plus (IPP) software (version 5.0.1, Media Cybernetics Inc., Rockville, MD). The average of the results was used for statistical analysis and expressed as mean optical density (MOD).

### Statistical analysis

Experiments were repeated in triplicate. All statistical analyses were performed using the SPSS^®^ statistical package, version 11.5 (SPSS Inc., Chicago, IL) for Windows^®^. Standard deviation and average were calculated, expressed as the mean ± SD values. The data were analyzed for significance using one-way analysis of variance (ANOVA), followed by the Student-Newman-Keuls test for multiple comparisons. A P-value of <0.05 was considered to indicate a statistically significant difference.

## Results

### AGE stimulation upregulates bFGF expression in Müller cells in a dose-dependent manner

#### Day 1

**Figure 1:**
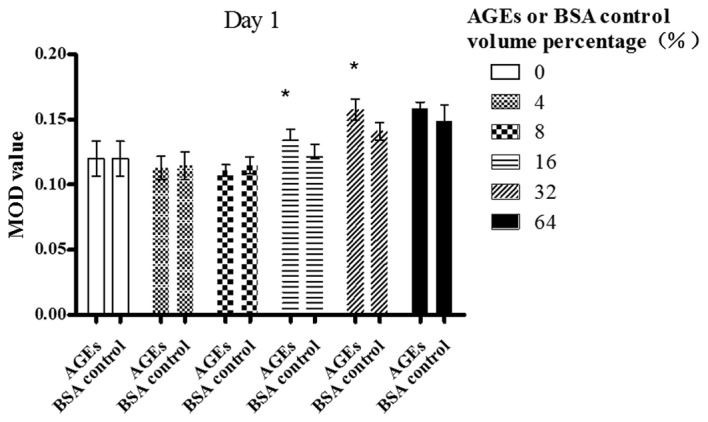
Effect of AGEs or BSA control on the production of bFGF by Müller cells (day 1). Müller cells were treated with 0, 8, 16, 32 and 64% volume percentage of AGEs or 0, 8 16, 32 and 64% volume percentages of non-glycated BSA (indicated on the abscissa) and the MOD value is indicated on the ordinate. ^*^p<0.05 compared with the BSA control.

A dose-dependent increase in the MOD value of bFGF was revealed following the exposure of Müller cells to AGE in a volume percentage ranging from 8 to 32%; however, the value did not change at 64%, compared with the value at 32% (P>0.05). There was a statistically significant difference between the cells treated with AGEs and those treated with the BSA control in a volume percentage of 16 and 32% (P<0.05) (Fig. 1).

#### Day 3

**Figure 2:**
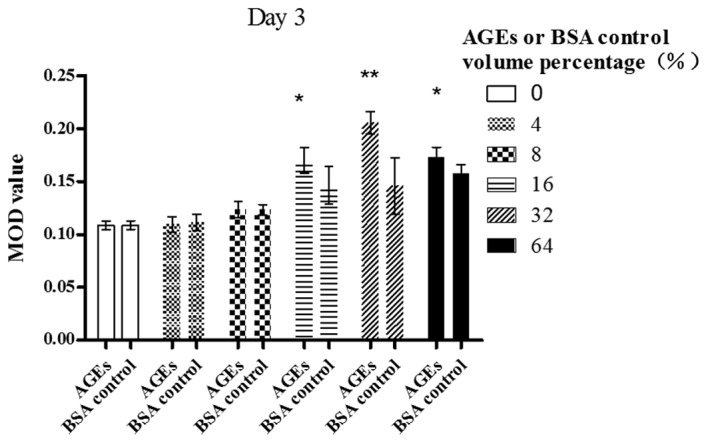
Effect of AGEs or BSA control on the production of bFGF by Müller cells (day 3). Müller cells were treated with 0, 8, 16, 32 and 64% volume percentages of AGEs or 0, 8, 16, 32 and 64% volume percentages of non-glycated BSA (indicated on the abscissa) and the MOD value is indicated on the ordinate. ^*^p<0.05 compared with the BSA control, ^**^p<0.01 compared with the BSA control.

A dose-dependent increase in the MOD value of bFGF was revealed following the exposure of Müller cells to AGE in a volume percentage ranging from 8 to 32%; however, the value began to decrease at 64% (P<0.01). AGEs (64% volume percentage) also significantly enhanced the bFGF expression in contrast with the blank control (P<0.01). There was a statistically significant difference between the cells treated with AGEs and those treated with the BSA control in a volume percentage of 16% (P<0.05), 32% (P<0.01) and 64% (P<0.05) (Fig. 2).

#### Day 6

**Figure 3:**
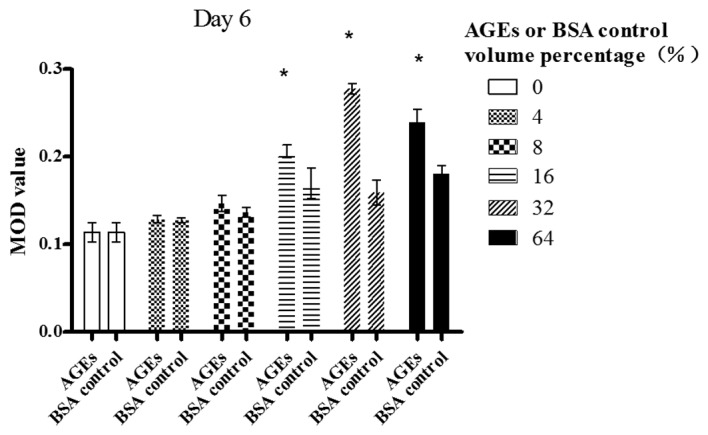
Effect of AGEs or BSA control on the production of bFGF by Müller cells (day 6). Müller cells were treated with 0, 8, 16, 32 and 64% volume percentages of AGEs or 0, 8, 16, 32 and 64% volume percentages of non-glycated BSA (indicated on the abscissa) and the MOD values are indicated on the ordinate. ^*^p<0.05 compared with the BSA control.

A dose-dependent increase in the MOD value bFGF was revealed following the exposure of Müller cells to AGE in a volume percentage ranging from 8 to 32%; however, the value began to decrease at 64% (P<0.01). AGEs (64% volume percentage) also significantly enhanced bFGF expression in contrast with the blank control (P<0.01). There was statistically significant difference between the cells treated with AGEs and those treated with the BSA control in a volume percentage of 16, 32 and 64% (P<0.05) (Fig. 3).

#### Day 9

**Figure 4:**
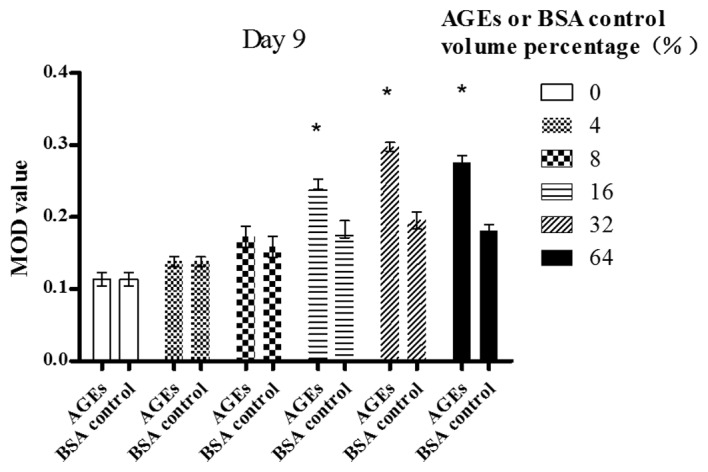
Effect of AGEs or BSA control on the production of bFGF by Müller cells (day 9). Müller cells were treated with 0, 8, 16, 32 and 64% volume percentages of AGEs or 0, 8, 16, 32 and 64% volume percentages of non-glycated BSA (indicated on the abscissa) and the MOD values are indicated on the ordinate. ^*^p<0.05 compared with the BSA control.

A dose-dependent increase in the MOD value of bFGF was revealed following the exposure of Müller cells to AGE in a volume percentage ranging from 0 to 32%; however, the value began to decrease significantly at 64% (P<0.05). AGEs (64% volume percentage) also significantly enhanced bFGF expression in contrast with the blank control (P<0.01). There was a statistically significant difference between the cells treated with AGEs and those treated with the BSA control in a volume percentage of 16, 32 and 64% (P<0.05) (Fig. 4).

### AGE stimulation upregulates bFGF expression in Müller cells in a time-dependent manner

**Figure 5:**
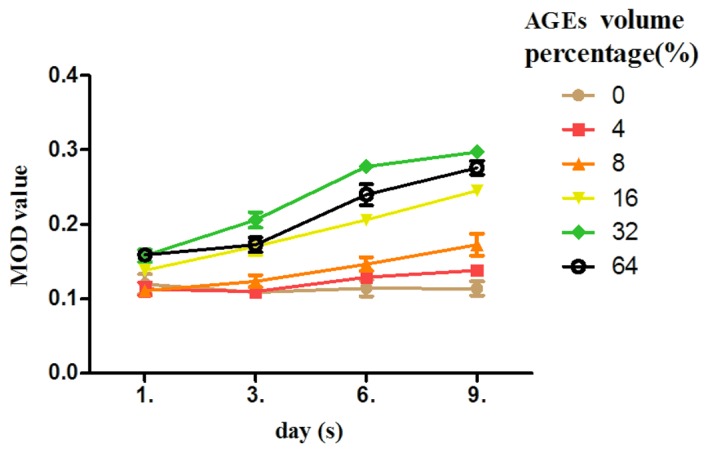
Effect of AGEs on the production of bFGF by Müller cells. Müller cells were treated with 0, 8, 16, 32 and 64% volume percentages of AGEs. The culture period after the addition of AGEs is indicated on the abscissa and the MOD values are indicated on the ordinate.

As shown in Fig. 5, at a volume percentage of 0% (the blank control), no statistically significant difference in the expression of bFGF was observed between the groups (F=1.229, P>0.05). At a volume percentage of 4%, the expression of bFGF increased from day 3 to 6 (P<0.01). At a volume percentage between 8 and 64%, the expression of bFGF increased from day 3 to 9 (P<0.05). At a volume percentage between 16 and 32%, the expression of bFGF increased from day 1 to 9 (P<0.01).

## Discussion

The accelerated formation and accumulation of AGEs has been implicated in the pathogenesis of diabetic vascular complications. AGEs directly or indirectly induce the production of various cytokines and growth factors by macrophages, monocytes, endothelial cells and Müller cells, which leads to the development of diabetic angiopathy (22–26).

Long-lasting retinal ischemia in DR causes the outgrowth of new vessels from superficial veins and venules onto the posterior vitreous cortex. The most severe ocular complications of diabetes mellitus are associated with proliferative DR (PDR). Once neovascularization develops, DR is classified as PDR. Various reactions may be discerned in the development of PDR, including chemotaxis and cellular migration, cellular proliferation, membrane formation and contraction (7). In eyes with PDR and persistent vitreoretinal adhesions, elevated neovascular fronds may undergo fibrous change and form tight fibrovascular bands that tug on the retina and exert continued vitreous contraction. This may cause a progressive traction retinal detachment.

Müller cells extend from the inner limiting membrane of the retina to the outer limiting membrane. It has been demonstrated that Müller cells are directly involved in the formation of the blood-retinal barrier (6,27) and grow specifically into the subretinal space and form multiple layers of cell bodies between the retina and the retinal pigment epithelium after retinal detachment. Therefore, Müller cells, in association with blood-derived immune cells and factors within the vitreous, are suggested to play a crucial role in either non-proliferative DR (NPDR) or PDR (28,29).

VEGF is deemed to be a part of a pro-survival response of the hypoxic tissue with actions that include vasodilatation, endothelial cell survival, inflammation, glial cell proliferation, neuroprotection, neurogenesis and neovascularization (30,31). In proliferative vitreous retinopathy (PVR) and PDR, the vitreal and subretinal concentration of VEGF is enhanced (32–34). bFGF is involved in many biological processes in embryonic development, wound healing, hematopoiesis and angiogenesis (35) and evokes the release of VEGF and HGF from Müller cells (36). These two cytokines (VEGF and bFGF) exert a synergistic effect during the several stages of angiogenesis in the retina (37).

In this study, we demonstrated that AGEs induce bFGF secretion by Müller cells in a dose- and time-dependent manner *in vitro*. However, when the maximum volume percentage was reached, bFGF expression tended to decrease, since bFGF has a down-regulatory effect on Müller cells.

As an angiogenic factor *in vivo*, bFGF is known to be more potent than VEGF and the formation of neovascularization is augmented by the accelerated flux through the bFGF secretion (38). Based on the present findings, we propose a reciprocal correlation between AGEs and bFGF expression. AGEs elicit the augmented secretion of bFGF in Müller cells, which synergistically promotes the development of diabetic vascular complications with VEGF. AGEs may thus affect the formation of retinal neovascularization directly, as well as indirectly, by the induction of bFGF expression; this therefore may be another relevant mechanism that leads to diabetic vascular dysfunction.

One limitation of our study is that there is no standard method of preparing AGE proteins and quantifying their chemical composition *in vitro.* A number of widely varying methods have been used for incubating cells with BSA. The composition of the final AGE-protein is largely unknown or not specified *in vivo*. Therefore, we used volume percentage to describe the concentrations, after we first measured the concentrations using a fluorescence spectrometer.

In conclusion, the increased formation of AGEs in the vitreous may be involved in the initiation and progression of intraocular neovascularization in diabetes through the production of bFGF by Müller cells. The results from our study are in accord with those from other studies suggesting that early vitrectomy in DR cases by clearing the AGEs and other molecules may contribute to prevent the progression of DR (7,15,33,39). Further *in vivo* studies are required to explore the importance of AGEs in the initiation and progression of DR and to develop new therapeutic strategies to prevent DR.

## References

[1] Daroux M, Prévost G, Maillard-Lefebvre H, Gaxatte C, D’Agati VD, Schmidt AM, Boulanger E (2010). Advanced glycation end-products: implications for diabetic and non-diabetic nephropathies. Diabetes Metab.

[2] Berrou J, Tostivint I, Verrecchia F, Berthier C, Boulanger E, Mauviel A, Marti HP, Wautier MP, Wautier JL, Rondeau E, Hertig A (2009). Advanced glycation end products regulate extracellular matrix protein and protease expression by human glomerular mesangial cells. Int J Mol Med.

[3] Wolffenbuttel BH, Giordano D, Founds HW, Bucala R (1996). Long-term assessment of glucose control by haemoglobin-AGE measurement. Lancet.

[4] Meerwaldt R, Links T, Zeebregts C, Tio R, Hillebrands JL, Smit A (2008). The clinical relevance of assessing advanced glycation endproducts accumulation in diabetes. Cardiovasc Diabetol.

[5] Yamagishi S (2011). Role of advanced glycation end products (AGEs) and receptor for AGEs (RAGE) in vascular damage in diabetes. Exp Gerontol.

[6] Bringmann A, Wiedemann P (2009). Involvement of Müller glial cells in epiretinal membrane formation. Graefes Arch Clin Exp Ophthalmol.

[7] Simó R, Carrasco E, García-Ramírez M, Hernández C (2006). Angiogenic and antiangiogenic factors in proliferative diabetic retinopathy. Curr Diabetes Rev.

[8] Aiello LP, Avery RL, Arrigg PG, Keyt BA, Jampel HD, Shah ST, Pasquale LR, Thieme H, Iwamoto MA, Park JE (1994). Vascular endothelial growth factor in ocular fluid of patients with diabetic retinopathy and other retinal disorders. N Engl J Med.

[9] Kakehashi A, Inoda S, Mameuda C, Kuroki M, Jono T, Nagai R, Horiuchi S, Kawakami M, Kanazawa Y (2008). Relationship among VEGF, VEGF receptor, AGEs and macrophages in proliferative diabetic retinopathy. Diabetes Res Clin Pract.

[10] Payne JF, Tangpricha V, Cleveland J, Lynn MJ, Ray R, Srivastava SK (2011). Serum insulin-like growth factor-I in diabetic retinopathy. Mol Vis.

[11] Shimizu F, Sano Y, Haruki H, Kanda T (2011). Advanced glycation end-products induce basement membrane hypertrophy in endoneurial microvessels and disrupt the blood-nerve barrier by stimulating the release of TGF-β and vascular endothelial growth factor (VEGF) by pericytes. Diabetologia.

[12] Simó R, Vidal MT, García-Arumí J, Carrasco E, García-Ramírez M, Segura RM, Hernández C (2006). Intravitreous hepatocyte growth factor in patients with proliferative diabetic retinopathy: a case-control study. Diabetes Res Clin Pract.

[13] Yang XM, Yafai Y, Wiedemann P, Kuhrt H, Wang YS, Reichenbach A, Eichler W (2012). Hypoxia-induced upregulation of pigment epithelium-derived factor by retinal glial (Müller) cells. J Neurosci Res.

[14] He S, Jin ML, Worpel V, Hinton DR (2003). A role for connective tissue growth factor in the pathogenesis of choroidal neovascularization. Arch Ophthalmol.

[15] Watanabe D, Suzuma K, Suzuma I, Ohashi H, Ojima T, Kurimoto M, Murakami T, Kimura T, Takagi H (2005). Vitreous levels of angiopoietin 2 and vascular endothelial growth factor in patients with proliferative diabetic retinopathy. Am J Ophthalmol.

[16] Cao R, Eriksson A, Kubo H, Alitalo K, Cao Y, Thyberg J (2004). Comparative evaluation of FGF-2-, VEGF-A- and VEGF-C-induced angiogenesis, lymphangiogenesis, vascular fenestrations and permeability. Circ Res.

[17] Tokuda H, Adachi S, Matsushima-Nishiwaki R, Kato K, Natsume H, Otsuka T, Kozawa O (2011). Enhancement of basic fibroblast growth factor-stimulated VEGF synthesis by Wnt3a in osteoblasts. Int J Mol Med.

[18] Lee JJ, Hsiao CC, Yang IH, Chou MH, Wu CL, Wei YC, Chen CH, Chuang JH (2012). High-mobility group box 1 protein is implicated in advanced glycation end products-induced vascular endothelial growth factor A production in the rat retinal ganglion cell line RGC-5. Mol Vis.

[19] Wakakura M, Foulds WS (1988). Immunocytochemical characteristics of Müller cells cultured from adult rabbit retina. Invest Ophthalmol Vis Sci.

[20] Liu Y, Wakakura M (1998). P1-/P2-purinergic receptors on cultured rabbit retinal Müller cells. Jpn J Ophthalmol.

[21] Neumann A, Schinzel R, Palm D, Riederer P, Münch G (1999). High molecular weight hyaluronic acid inhibits advanced glycation endproduct-induced NF-kappaB activation and cytokine expression. FEBS Lett.

[22] Vlassara H, Brownlee M, Manogue KR, Dinarello CA, Pasagian A (1988). Cachectin/TNF and IL-1 induced by glucose-modified proteins: role in normal tissue remodeling. Science.

[23] Walcher D, Marx N (2009). Advanced glycation end products and C-peptide-modulators in diabetic vasculopathy and atherogenesis. Semin Immunopathol.

[24] Yamagishi S, Maeda S, Matsui T, Ueda S, Fukami K, Okuda S (2012). Role of advanced glycation end products (AGEs) and oxidative stress in vascular complications in diabetes. Biochim Biophys Acta.

[25] Nam MH, Lee HS, Seomun Y, Lee Y, Lee KW (2011). Monocyte-endothelium-smooth muscle cell interaction in co-culture: proliferation and cytokine productions in response to advanced glycation end products. Biochim Biophys Acta.

[26] Curtis TM, Hamilton R, Yong PH, McVicar CM, Berner A, Pringle R, Uchida K, Nagai R, Brockbank S, Stitt AW (2011). Müller glial dysfunction during diabetic retinopathy in rats is linked to accumulation of advanced glycation end-products and advanced lipoxidation end-products. Diabetologia.

[27] Bhatia B, Jayaram H, Singhal S, Jones MF, Limb GA (2011). Differences between the neurogenic and proliferative abilities of Müller glia with stem cell characteristics and the ciliary epithelium from the adult human eye. Exp Eye Res.

[28] Guidry C, King JL, Mason JO (2009). Fibrocontractive Müller cell phenotypes in proliferative diabetic retinopathy. Invest Ophthalmol Vis Sci.

[29] King JL, Mason JO, Cartner SC, Guidry C (2011). The influence of alloxan-induced diabetes on Müller cell contraction-promoting activities in vitreous. Invest Ophthalmol Vis Sci.

[30] Crawford TN, Alfaro DV, Kerrison JB, Jablon EP (2009). Diabetic retinopathy and angiogenesis. Curr Diabetes Rev.

[31] Liu W, Xu J, Wang M, Wang Q, Bi Y, Han M (2011). Tumor-derived vascular endothelial growth factor (VEGF)-a facilitates tumor metastasis through the VEGF-VEGFR1 signaling pathway. Int J Oncol.

[32] Dieudonné SC, La Heij EC, Diederen RM, Kessels AG, Liem AT, Kijlstra A, Hendrikse F (2007). Balance of vascular endothelial growth factor and pigment epithelial growth factor prior to development of proliferative vitreoretinopathy. Ophthalmic Res.

[33] Funatsu H, Noma H, Mimura T, Eguchi S, Hori S (2009). Association of vitreous inflammatory factors with diabetic macular edema. Ophthalmology.

[34] Baharivand N, Zarghami N, Panahi F, Dokht Ghafari MY, Mahdavi Fard A, Mohajeri A (2012). Relationship between vitreous and serum vascular endothelial growth factor levels, control of diabetes and microalbuminuria in proliferative diabetic retinopathy. Clin Ophthalmol.

[35] Kanda S, Naba A, Miyata Y (2009). Inhibition of endothelial cell chemotaxis toward FGF-2 by gefitinib associates with downregulation of Fes activity. Int J Oncol.

[36] Hollborn M, Jahn K, Limb GA, Kohen L, Wiedemann P, Bringmann A (2004). Characterization of the basic fibroblast growth factor-evoked proliferation of the human Müller cell line, MIO-M1. Graefes Arch Clin Exp Ophthalmol.

[37] Zakareia FA, Alderees AA, Al Regaiy KA, Alrouq FA (2010). Correlation of electroretinography b-wave absolute latency, plasma levels of human basic fibroblast growth factor, vascular endothelial growth factor, soluble fatty acid synthase and adrenomedullin in diabetic retinopathy. J Diabetes Complications.

[38] Matsui M, Tabata Y (2012). Enhanced angiogenesis by multiple release of platelet-rich plasma contents and basic fibroblast growth factor from gelatin hydrogels. Acta Biomater.

[39] Gündüz K, Bakri SJ (2007). Management of proliferative diabetic retinopathy. Compr Ophthalmol Update.

